# CRISPR Taking the Front Seat in Immunotherapy

**DOI:** 10.33696/immunology.2.032

**Published:** 2020

**Authors:** Saad S. Kenderian, Andrew D. Badley

**Affiliations:** 1 T Cell Engineering, Mayo Clinic, Rochester, MN, USA; 2 Division of Hematology, Mayo Clinic, Rochester, MN, USA; 3 Department of Immunology, Mayo Clinic, Rochester, MN, USA; 4 Department of Molecular Medicine, Mayo Clinic, Rochester, MN, USA; 5 Division of Infectious Diseases, Mayo Clinic, Rochester, MN, USA

## Introduction

CRISPR (clustered regularly interspaced short palindromic repeats) technology has dramatically simplified genome editing and is widely applicable in both basic research and therapeutic areas. The basic principle of CRISPR relies on the use of guide RNA which is designed to bind to the DNA sequence of interest along with a CRISPR-associated (Cas) endonuclease which introduces a double-stranded DNA break (DSB) at that site. Prior to the advent of the CRISPR revolution within the last decade, gene editing was dependent on predicting and engineering protein-DNA interactions mediated by zinc-finger nucleases (ZFNs) and transcription activator-like effector nucleases (TALENs). In contrast, CRISPR relies on simple Watson-Crick base pairing between the guide RNA and the target DNA sequence. The simplicity and versatility of CRISPR has led to its widespread implementation by the scientific community and subsequent unparalleled progress in genetic engineering.

## Mechanisms of CRISPR Induced DNA Breaks

Since the first report of CRISPR-edited human cells in 2013 [1], CRISPR systems have been used to both knock out and knock in genes. Gene knockouts are created through the nonhomologous end-joining (NHEJ) pathway, which rapidly ligates the ends of the DSB without a DNA repair template [2]. NHEJ is error-prone and frequently introduces small indels into the gene, most often leading to frameshift mutations and loss-of-function of the target gene, although NHEJ has also been used to restore function in genes with existing frameshift mutations [3]. Alternatively, gene knock-ins can be achieved through homology-directed repair (HDR), a separate pathway which uses a DNA template with long homologous sequences to repair the DNA. Instead of using the sister chromatid as a repair template, researchers have exploited HDR to generate CRISPR-mediated gene knock-ins by including an exogenous DNA template with the desired transgene flanked by sequences homologous to either end of the DSB [3,4]. Microhomology-mediated end-joining (MMEJ) is an alternate pathway which relies on short homology arms to repair the DSB [5]. Though the mechanisms of MMEJ are not as well understood, MMEJ can also be used to knock in genes using CRISPR technology [6,7]. MMEJ-mediated knock-in strategies appear experimentally similar to those used for HDR, but the use of shorter homology arms allows for easier cloning of the knockin template [6,7].

## Application of CRISPR in Immunotherapy

In their review article in the May 2019 edition of *Haematologica,* González-Romero et al. [8] discuss the uses of CRISPR-Cas9 systems in hematological diseases and succinctly outline the uses of CRISPR in the modification of chimeric antigen receptor T (CART) cells. CARs are synthetic receptors comprised of an extracellular single chain variable fragment of an antibody, hinge region and transmembrane domain, and intracellular signaling domains derived from the T cell receptor (TCR). The CAR redirects and activates T cells against cancer cells which express the cognate antigen. The authors briefly discussed various CRISPR applications in CART therapy and concluded that the combination of CRISPR and CART will fine-tune the engineered T cells and advance the field of cancer immunotherapy. Indeed, CRISPR-modified CART cells have shown to be safer and have better antitumor efficacy compared to traditionally manufactured CART cells in preclinical settings, and several clinical trials are underway (Table 1) [9-15].

Scientists have been using genome editing technology to create universal CART cells for nearly a decade. Knocking out the TCR and human leukocyte antigen (HLA) molecules are key to mitigating both the risk of graft-versus-host disease (GvHD) and the possibility of graft rejection of allogeneic cell products [10,16]. In 2012, researchers used ZFNs to knock out TCRα and TCRβ constant (TRAC and TRBC) chains in CART cells and demonstrated that these TCR-knockout CART cells only responded to stimulation through the CAR and did not cause GvHD in mice [17]. The following year, the group used the same approach to knock out the HLA-A gene to circumvent graft rejection of the engineered T cells; HLA-A-knockout T cells evaded killing by HLA-restricted T cells from a different donor and also avoided natural killer cell immunosurveillance by expressing nonclassical HLA molecules [17]. Similarly, TALEN technology was used to disrupt TRAC and CD52 genes in CART cells to prevent GvHD and to render the CART product resistant to elimination by the anti-CD52 lymphodepleting agent, alemtuzumab. This CART product was taken to the clinic and resulted in the complete remission and subsequent successful allogeneic stem cell transplantation of two infants with B-cell acute lymphoblastic leukemia [18].

Given the promising preclinical and clinical results in genome-edited CART cells, many groups have used CRISPR to knock out one or more endogenous genes in CART cells. These studies generally aim to 1) enhance CART efficacy by knocking out genes which lead to T cell exhaustion [9,13,15], 2) create an off-the-shelf allogeneic CART product by knocking out TCR and HLA components [19], 3) allow CART cells to attack malignant T cells without the risk of fratricide by knocking out the CAR target on the CART cell [20], or 4) improve CART safety profiles by knocking out genes which contribute to adverse side effects associated with CART therapy [21].

Researchers have sought to create allogeneic, exhaustion-resistant CART cells by combining lentiviral transduction of the CAR transgene with electroporation of Cas9 mRNA and guide RNA targeted to PD1, TCR, and HLA gene loci. These modified CART cells demonstrated reduced alloreactivity *in vitro* and improved antitumor effects in both hematological and solid tumor xenograft models which express the PD1 ligand PD-L1 [22]. The same group employed a multiplex genome editing platform by including multiple guide RNA cassettes with the CAR transgene in a lentiviral vector, allowing for simultaneous knockout of up to four genes in the CART cells after a single electroporation of Cas9 mRNA [23]. Triple knockout of PD1, TCR, and HLA molecules in CART cells also prolonged survival in a mouse glioblastoma model [9]. CRISPR-mediated knockout of checkpoint molecules such as PD1 may improve CART activity especially in solid tumors, an area in which the CART field has seen limited success in part due to the immunosuppressive tumor microenvironment. Several clinical trials in China are ongoing in testing CRISPR-edited CART cells with knockout of immune checkpoint molecules such as PD1 (NCT03747965, NCT03545815) and HPK1 (NCT04037566) as well as universal CART cells (NCT03166878, NCT03398967) (Table 2). CRISPR-CART clinical trials also have been initiated in the US to test allogeneic CART cells (NCT04035434) (Table 2). Until recently, CART therapy could not be used to treat cancers such as T-cell acute lymphoblastic leukemia, as the CARs would be activated against T cell antigens expressed on both the malignant T cells and the CART cells themselves. Scientists have used CRISPR to produce CD7-targeting CART cells with CD7 knocked out, rendering them cytotoxic to endogenous T cells but resistant to their own CAR; a clinical trial has been initiated in the US to test this platform in patients with a variety of T cell malignancies (NCT03690011) (Table 2).

Scientists have also used CRISPR-edited CART cells to prevent or reduce cytokine release syndrome (CRS) and neurotoxicity, the two most common severe side effects of CART therapy [21]. Granulocyte-macrophage colony-stimulating factor (GM-CSF) is critical to monocyte modulation, which in turn plays a role in the development of both CRS and neurotoxicity. Our group has shown that GM-CSF neutralization prevents the development of CRS and leads to significant reduction of neurotoxicity. Additionally, using CRISPR-Cas9 to knock out GM-CSF in CART cells had no adverse effects on normal CART cell function, and these knockout CART cells also displayed superior anti-leukemic activity and prolonged overall survival *in vivo* compared to wild-type GM-CSF CART cells [14,21]. Interestingly, the improved antitumor efficacy was observed in an immunocompromised mouse model with an absence of myeloid cells, indicating direct effects of GM-CSF knockout on CART cells in addition to the impact on myeloid cells. Further analysis uncovered a link between GM-CSF knockout and inhibition of the Fas death pathway [24]. CRISPR technology has enabled the production of CART cells that are both safer and more effective in preclinical studies and presents an exciting opportunity for bringing improved CART products to patients.

Most studies to date have used CRISPR to knock out undesired genes in CART cells, but CRISPR has also been used to knock in the CAR at a specific locus in the T cell genome [19]. CRISPR-Cas9 and CAR transgenes have been combined in a lentiviral vector to knock in the CAR transgene at the TRAC locus, resulting in simultaneous TCR knockout. These CART cells had stronger antileukemic activity in a mouse xenograft model [19]. An alternate system, CRISPR-Cas12a, was used in combination with CARs in adeno-associated viral vectors to generate double knock-in/knock-outs with a CD19 CAR at the PD1 locus and a CD22 CAR at the TRAC locus [25]. Interestingly, Cas12a was more effective at producing double knock-in/knock-outs than the more commonly used CRISPR-Cas9 platform, and CRISPR-Cas12a-edited CART cells expressed lower levels of exhaustion markers than CRISPR-Cas9-edited CART cells [25]. Furthermore, targeted CAR delivery creates a more uniform cellular product, in sharp contrast with the random integration of viral transduction methods most commonly used to generate CART cells [25]. CART therapy is expensive and highly variable, and specific CAR integration into a known genomic locus will reduce batch-to-batch variation. In turn, more uniform CART products will likely diminish the regulatory cost and burden involved in producing current CART products as well as in approving new CART therapies. Insertional oncogenesis, although never observed with modified T cells in the clinic to date, remains a possibility with any therapy involving a non-targeted, integrated transgene; CAR integration at a known genomic site would alleviate these concerns as well.

As stated in the review by González-Romero et al. [8], it would be “hard to underestimate” the impact of CRISPR technology paired with CART cells. Great strides have been taken to improve both the function and safety profile of CART cells, as well as in creating universal allogeneic CART cells to overcome the logistical and economic hurdles presented by current CART manufacturing techniques [9,10,13,14,19,22,23]. However, there are many areas that need additional exploration. Cas9 is the CRISPR system most commonly implemented in genome editing, but several other CRISPR variants are also viable candidates, most notably CRISPR-Cas12a. Cas12a recognizes a different protospacer adjacent motif than Cas9, broadening the potential target sequences. Instead of creating blunt ends at the DSB as seen in Cas9, Cas12a generates sticky ends which are less likely to be repaired by NHEJ and may be preferable to HDR-mediated gene knock-ins [25]. Direct comparisons of Cas9 and Cas12a systems are largely lacking to date. The majority of studies have used various viral vectors to edit the T cell genome, but when translating CART to a clinical and commercial product, GMP-grade virus presents a huge expense and logistical challenge. One report described the generation of CRISPR-edited TCR-engineered T cells using completely nonviral methods [26]. More research is needed in the production of nonviral CRISPR-edited CART cells to overcome current manufacturing challenges of clinical-grade CART products. Finally, CRISPR is most often used to enhance the therapeutic efficacy of CART cells, but it can also be a valuable screening tool. One such study used CRISPR gene knockout screens to investigate the mechanisms behind the effects of various small molecule drugs on CART cytotoxicity [27]. CRISPR libraries can enhance our understanding of CART interactions with existing drugs and influence choices made in the clinic regarding CART and chemotherapeutic drug combinations. Broadening the scope from CART cells, a recent study used a genome-wide CRISPR screen to discover an unconventional TCR that appears to be cancer-specific and HLA-independent [11]. Whether these unconventional T cells can be used as a standalone therapeutic or as a complement to traditional cancer treatments or alongside CART therapy remains to be seen, but this study highlights the importance of CRISPR technology in the discovery of additional immunotherapies. CRISPR has been used to enhance existing therapies, but it may also uncover entirely new avenues of cellular immunotherapy. CRISPR has proven to be a valuable tool in preclinical studies of CART cells, and clinical trials of CRISPR-edited CART cells will yield more information on utilizing this versatile genome editing technique to improve cancer immunotherapies and patient outcomes.

## Conclusion

In summary, the advances in CRISPR technology over the last decade have started to revolutionize cellular immunotherapy for the treatment of cancer. We have witnessed CRISPR genome engineering moving from “proof of concept” experiments to first in human clinical trials.

## Figures and Tables

**Table 1:** Preclinical CRISPR-edited CART studies.

Objective	CRISPR System	Delivery Methods	Targets	Reference
Universal, exhaustion-resistant	Cas9	Electroporation of Cas9 mRNA and guide RNA + lentiviral CD19− or PSCA-CAR	TCR, HLA, PD1	Ren et al. [22]
Universal, exhaustion-resistant	Cas9	Electroporation of Cas9 mRNA + lentiviral CD19− or PSCA-CAR and guide RNA cassettes	TCR, HLA, PD1, CTLA-4	Ren et al. [23]
Universal, exhaustion-resistant	Cas9	Electroporation of Cas9/guide RNA RNPs + AAV EGFRvIII-CAR	TCR, HLA, PD1	Choi et al. [9]
Prevention or reduction of CRS and neurotoxicity	Cas9	Electroporation of Cas9/guide RNA RNPs + lentiviral CD19-CAR	GM-CSF	Sterner et al. [21]
Targeted CAR knockin	Cas9	Electroporation of Cas9 mRNA and guide RNA + AAV CD19-CAR with homology arms	TCR (CAR knockin)	Eyquem et al. [19]
Targeted dual CAR knockin	Cas12a	Electroporation of Cas12a mRNA + lentiviral CD19− or CD22-CAR with homology arms and guide RNA cassettes	TCR, PD1 (CAR knockins)	Dai et al. [25]

**Table 2:** Initiated and ongoing clinical trials of CRISPR-edited CART cells.

Objective	CAR Target	CRISPR Target	Clinical Trial Identifier
Exhaustion-resistant	Mesothelin	PD1	NCT03747965
Exhaustion-resistant	CD19	HPK1	NCT04037566
Exhaustion-resistant, universal	Mesothelin	PD1, TCR	NCT03545815
Allogeneic	CD19	TCR, HLA	NCT04035434
Allogeneic	CD19	TCR, HLA	NCT03166878
Allogeneic	CD19/CD20 or CD19/CD22	TCR, HLA	NCT03398967
Target T cell malignancies	CD7	CD7	NCT03690011
